# Two Distinct *Yersinia pestis* Populations Causing Plague among Humans in the West Nile Region of Uganda

**DOI:** 10.1371/journal.pntd.0004360

**Published:** 2016-02-11

**Authors:** Laurel B. Respicio-Kingry, Brook M. Yockey, Sarah Acayo, John Kaggwa, Titus Apangu, Kiersten J. Kugeler, Rebecca J. Eisen, Kevin S. Griffith, Paul S. Mead, Martin E. Schriefer, Jeannine M. Petersen

**Affiliations:** 1 Centers for Disease Control and Prevention, Division of Vector-Borne Diseases, Bacterial Diseases Branch, Fort Collins, Colorado, United States of America; 2 Uganda Virus Research Institute, Entebbe, Uganda; University of Tennessee, UNITED STATES

## Abstract

**Background:**

Plague is a life-threatening disease caused by the bacterium, *Yersinia pestis*. Since the 1990s, Africa has accounted for the majority of reported human cases. In Uganda, plague cases occur in the West Nile region, near the border with Democratic Republic of Congo. Despite the ongoing risk of contracting plague in this region, little is known about *Y*. *pestis* genotypes causing human disease.

**Methodology/Principal Findings:**

During January 2004–December 2012, 1,092 suspect human plague cases were recorded in the West Nile region of Uganda. Sixty-one cases were culture-confirmed. Recovered *Y*. *pestis* isolates were analyzed using three typing methods, single nucleotide polymorphisms (SNPs), pulsed field gel electrophoresis (PFGE), and multiple variable number of tandem repeat analysis (MLVA) and subpopulations analyzed in the context of associated geographic, temporal, and clinical data for source patients. All three methods separated the 61 isolates into two distinct 1.ANT lineages, which persisted throughout the 9 year period and were associated with differences in elevation and geographic distribution.

**Conclusions/Significance:**

We demonstrate that human cases of plague in the West Nile region of Uganda are caused by two distinct 1.ANT genetic subpopulations. Notably, all three typing methods used, SNPs, PFGE, and MLVA, identified the two genetic subpopulations, despite recognizing different mutation types in the *Y*. *pestis* genome. The geographic and elevation differences between the two subpopulations is suggestive of their maintenance in highly localized enzootic cycles, potentially with differing vector-host community composition. This improved understanding of *Y*. *pestis* subpopulations in the West Nile region will be useful for identifying ecologic and environmental factors associated with elevated plague risk.

## Author Summary

Plague, a severe and often fatal zoonotic disease, is caused by the bacterium *Yersinia pestis*. Currently, the majority of human cases have been reported from resource limited areas of Africa, where the proximity to commensal rats and other small mammals increases the likelihood for human contact with infected animals or their fleas. Over a 9 year time period, >1000 suspect cases were recorded in the West Nile region of Uganda within the districts of Arua and Zombo. Culture-confirmed cases were shown by three independent typing methods to be due to two distinct 1.ANT genetic subpopulations of *Y*. *pestis*. The two genetic subpopulations persisted throughout the 9 year time period, consistent with their ongoing maintenance in local enzootic cycles. Additionally, the two subpopulations were found to differ with respect to geographic location and elevation, with SNP Group 1 strains being found further north and at lower elevations as compared to SNP Group 2. The relative independence of the two *Y*. *pestis* subpopulations is suggestive of their maintenance in distinct foci involving enzootic cycles with differing vector-host community composition.

## Introduction

*Yersinia pestis* is the etiological agent of plague, a severe and often fatal disease in humans [1]. Transmission of *Y*. *pestis* to humans most often occurs through the bite of an infectious flea or by direct exposure to an infected mammalian host. Less frequently, human infection is the result of inhaling infectious respiratory droplets. The three primary clinical forms of human disease are bubonic, septicemic, and pneumonic plague. Bubonic plague is the most common and is characterized by one or more swollen and painful lymph nodes (buboes), while pneumonic plague is the most severe, with fatality rates approaching 100% in patients with untreated disease [2]. In nature, *Y*. *pestis* is maintained by various small mammal hosts and their associated fleas. Epizootics among susceptible mammals often precede human plague cases. The high host mortality forces infected fleas to bite alternate hosts, including humans [3].

The geographic distribution of plague is widespread with foci in the Americas, Africa, and Asia [1]. In recent decades, the majority of human cases have been reported from East Africa (Uganda, Tanzania, Democratic Republic of Congo) and Madagascar, in resource limited areas, where the proximity to commensal rats and other small mammals increases the likelihood for human contact with infected animals or their fleas [1,4,5,6]. In Uganda, the current plague focus encompasses an area of approximately 900 km^2^ in the West Nile region, situated above the Rift Valley escarpment and within the districts of Arua and Zombo [7,8]. Human cases in this region are concentrated in the counties of Okoro and Vurra and occur primarily between the months of September and December each year [9]. For the time period of 1999–2007, an incidence rate of >5 cases per 1,000 individuals was reported [7]. The risk for plague in this region is greater at elevations above 1,300 meters, and positively correlates with higher amounts of rainfall as compared to lower elevations. Ecologic differences above 1,300 meters compared with below include an increase in both abundance and diversity of small mammals and a greater diversity of flea species on mammalian hosts [4,8–11].

*Y*. *pestis* genotypes that cause human disease in the West Nile region of Uganda have not been previously described. Worldwide, limited nucleotide variation is observed between strains and *Y*. *pestis* is considered a genetically monomorphic pathogen [12]. Traditionally, three biovars of *Y*. *pestis*, antiqua, medievalis, and orientalis, have been differentiated based on their ability to metabolize glycerol or reduce nitrate [13]. With the advent of the genomic era and a rise in the number of sequenced *Y*. *pestis* genomes, single nucleotide polymorphisms (SNPs) have been used as the basis for defining worldwide populations of *Y*. *pestis* [14]. A global SNP analysis, based on 17 whole genome sequences to discover SNPs, demonstrated that *Y*. *pestis* strains separate into several populations with distinctive geographic patterns, including 1.ORI (orientalis; North and South America, Madagascar, Southeast Asia), 2.MED (mediaevalis; Asia), 1.ANT (antiqua; East and Central Africa), and 2.ANT (antiqua; Asia) [14].

Although SNPs are useful phylogenetic markers, their discovery from comparison of a limited number of whole genome sequences has only limited resolving power. For higher level differentiation, other mutation types have been exploited in *Y*. *pestis* genomes, including tandem repeats, insertion sequence (IS) mediated rearrangements, clustered regularly interspaced short palindromic repeats (CRISPRs), and insertions/deletions (INDELs) [15–18]. Strain typing methods capitalizing on these mutations include multi-locus variable number of tandem repeat (VNTR) analysis (MLVA), pulsed field gel electrophoresis (PFGE), CRISPR genotyping, and restriction fragment linked polymorphism (RFLP) of IS elements [15,17,19–22].

Here, three molecular typing methods detecting different genome mutations, SNPs, MLVA, and PFGE, were used to characterize 61 *Y*. *pestis* isolates recovered from human plague patients in the West Nile region of Uganda over a 9 year time span from 2004–2012. Identified subpopulations were analyzed in the context of associated geographic, temporal, and clinical data for source patients.

## Materials and Methods

### Ethics statement

*Y*. *pestis* isolates (not de-linked from originating patients) were cultured from samples collected with documented informed consent during three research studies approved by institutional review boards at the U.S. Centers for Disease Control and Prevention (CDC), the Uganda Virus Research Institute, and the Uganda National Council for Science and Technology. A small number of archived clinical isolates banked for public health purposes were also included.

### Location and bacterial strains

In total, 61 *Y*. *pestis* isolates were cultured from human plague cases occurring in Vurra and Okoro counties within the Arua and Zombo districts, respectively. This included the previously sequenced UG05-0454 strain, which was isolated from a patient in the West Nile region in 2004 [14]. For isolation of cultures, patient specimens (blood, bubo aspirate or sputum) were plated on 6% sheep blood agar (SBA) or cefsulodin-irgasan-novobiocin (CIN) agar and incubated at 35°C or 25°C, respectively. Plates were checked daily for growth and *Y*. *pestis* isolates identified by direct fluorescence antibody (DFA), polymerase chain reaction (PCR), and bacteriophage lysis [23]. For isolation of DNA, isolates were grown on 6% SBA at 35°C for 24 hrs and DNA isolated using the QIAmp DNA Mini Kit (Qiagen, Hilden, Germany). Clinical and demographic data on source patients was collected as part of ongoing surveillance and diagnosis of plague in the region. Extracted data included patient age, sex, illness onset date, form of clinical disease, and illness outcome. GPS coordinates for each patient’s residence were recorded as the likely exposure site.

### SNP analysis

A total of 9 SNPs, previously identified from whole genome sequencing of the *Y*. *pestis* strain UG05-0454, were selected for genotyping [14]. Melt mismatch amplification mutation assays (Melt-MAMA) were developed for each SNP [24–26]. Ancestral, derived, and consensus primer sequences for each assay are listed in S1 Table. All Melt-MAMA reactions were performed in a 10 μl final volume and contained SYBR green PCR Master Mix (Applied Biosystems, Foster City, CA) at a 1X final concentration, a common reverse primer (200 nM), derived and ancestral allele-specific MAMA primers (200 nM), water, and 1 μl of diluted template (approximately 2 ng DNA). Melt-MAMA assays were performed on an Applied Biosystems 7500 Fast Dx real-time PCR system using SDS software v1.4 and the following cycling conditions: 50°C for 2 min, 95°C for 10 min, then 95°C for 15 s and 55°C (s447: CO92 gene *ypo0064*) or 60°C (all others) for 1 min for 33 cycles followed by melt curve analysis using a dissociation protocol of 95°C for 15 s, then temperature ramping in 0.2°C/min increments from 60°C to 95°C.

### Pulsed Field Gel Electrophoresis

PFGE was performed with AscI enzyme using the standardized PulseNet protocol (http://www.pulsenetinternational.org/protocols). Isolates were grown on 6% SBA at 35°C for 24 hrs. Cells were resuspended in buffer (100 mM Tris:100 mM EDTA) at an optical cell density of 0.45–0.50 and agarose plugs prepared. To lyse cells, plugs were incubated in 50 mM Tris:50 mM EDTA, 0.1mg/ml Proteinase K, 1% sarcosyl for 2 hrs at 55°C, then washed in sterile water followed by TE buffer. DNA in embedded plugs was digested for 5 hrs with 40 U of AscI enzyme (New England Biolabs, Ipswich, MA) at 37°C and PFGE performed for 17.5 hrs at 14°C. *Salmonella enterica* serotype Braenderup (H9812) cut with 50 U XbaI enzyme (Roche Diagnostics, Indianapolis, IN) was used as a reference standard. PFGE parameters were 6 V/cm, 120°C, linear ramping, and switch times of 1.79 s-18.66 s. Gels were stained with ethidium bromide and imaged using a Gel Doc 1000 (BioRad, Hercules, CA). PFGE patterns were analyzed using BioNumerics software Version 6.64 (Applied Maths, Inc., Sint-Martens-Latem, Belgium) and patterns normalized to the reference standard. Cluster analysis of PFGE patterns was performed using Dice similarity coefficients (1.0% optimization and 1.5% tolerance) and unweighted pair group method with averages (UPGMA).

### Mutation rate estimate—*Asc*I loci

Mutation rate estimates for *Y*. *pestis Asc*I PFGE loci were determined from a parallel, single passage experiment [25, 26]. Briefly, a single colony of UG09-2299 was used to generate an *in vitro* population representing ~10,000 generations. The generation rate was determined by preparing 10 fold serial dilutions from a single *pgm*^+^ colony resuspended in 1 ml of PBS, plating dilutions to 6% SBA plates and counting colonies after growth for 48 hrs at 35°C. After growth for 48 hrs at 35°C, 100 colonies were selected and streaked to 100 SBA plates to represent passage 1. This process was repeated 3 more times and PFGE performed on the 100 clones from the final passage. Congo Red plates were included at all steps to verify the presence of the pigmentation (*pgm)* locus. The mutation rate was determined using the following: number of clonal lineages x number of passages x generations/colony.

### MLVA

Eighteen VNTR markers located across the *Y*. *pestis* genome, with previously determined *in vitro* mutation rates, were chosen [15,27,28]. PCR reactions were performed using PuReTaq Ready-To-Go PCR beads (GE Healthcare Life Sciences, Pittsburgh, PA) and the following cycling conditions: 94°C for 5 min, 35 cycles of 94°C for 20 s, 65°C for 20 s, and 72°C for 30 s, then 72°C for 5 min. PCR products were visualized on a QIAxcel system (Qiagen, Hilden, Germany) using the QIAxcel DNA High Resolution cartridge. VNTR amplicon sizes were calculated using BioCalculator 3.2 software (Qiagen, Hilden, Germany) and the QX reference alignment marker 15-500bp and QX DNA size marker 25-500bp (Qiagen, Hilden, Germany). Amplicon size and repeat length was used to determine the number of motifs in each allele. A correction factor was calculated for each marker using the product size estimated for the *Y*. *pestis* reference control strain CO92 (1.ORI; North America) and the known size for the loci in the CO92 genome. Repeat copy numbers for each loci in each strain were determined by applying the correction factor to the product size determined by the QIAxcel. The resulting data was imported into BioNumerics software Version 6.64 and cluster analysis performed using categorical coefficients and UPGMA.

### Statistical analyses and maps

Epidemiologic analyses were performed with SAS, version 9.3 (SAS Institute, Cary, NC). χ^2^ tests were used for categorical data; Wilcoxon rank-sum tests were used to compare continuous data. Analyses were considered to be statistically significant when P < .05. Maps were generated with the use of ArcMap, version 10.3 (ESRI, Redlands, CA) by plotting isolates based on global positioning systems (GPS) coordinates of patient residence.

## Results

### Human cases

**Fig 1:**
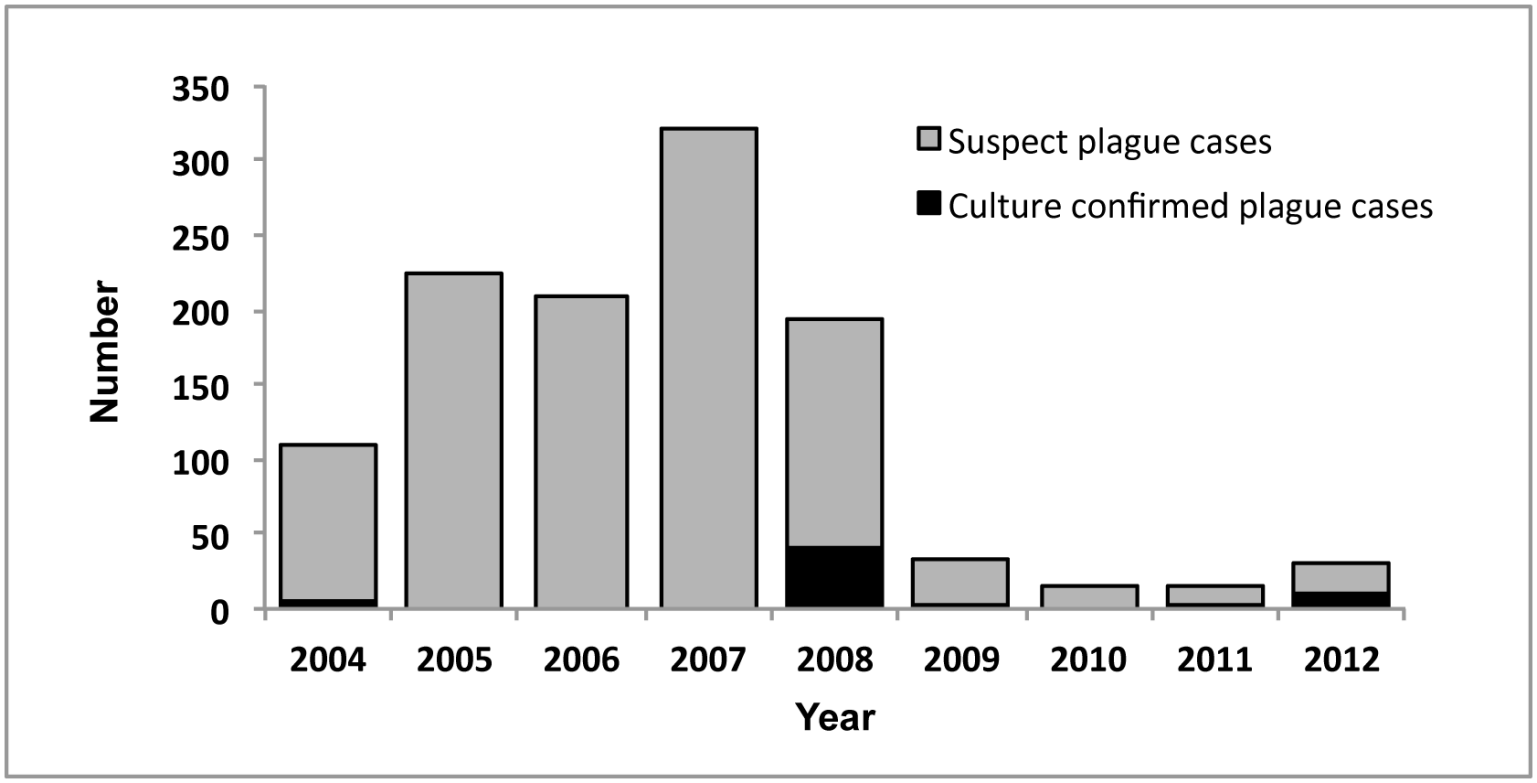
Human plague cases West Nile region of Uganda 2004–2012. Suspect (gray) and culture confirmed (black) cases are indicated for the time period from January 2004 through December 2012.

During January 2004—December 2012, a total of 1,092 suspect human plague cases were recorded in the West Nile region of Uganda, with a high in 2007 of 321 cases and a low in 2011 of 14 cases (Fig 1). Overall, 61 cases (<10% of suspect cases) were culture confirmed during this time period; 5 from 2004 (8%), 45 from 2008 (74%), 1 from 2009 (2%), 2 from 2011 (3%) and 8 from 2012 (13%) (Fig 1).

### SNPs

Based on unique SNPs previously identified in the UG05-0454 strain [14], nine melt-MAMA assays were developed and used to screen the 61 *Y*. *pestis* isolates (Table 1 and S2 Table). Across all isolates, four SNPs (s376, s1427, s357 and s1333) were the same as UG05-0454, indicating all 61 *Y*. *pestis* isolates fall into the 1.ANT lineage. One of these SNPs, s1333, was conserved across 58 isolates, but for 3 isolates this SNP could not be determined due to multiple failed amplification attempts (S2 Table). Three of the SNPs, s949, s860, and s968, separated the 61 *Y*. *pestis* isolates into 2 genetic groups, termed Group 1 and Group 2, with 22 (36%) and 39 (64%) strains comprising Group 1 and 2, respectively. The remaining two SNPs, s272 and s447, were unique to UG05-0454.

**Table 1:** SNP genotypes in the West Nile region of Uganda.

	SNP^a^									
SNP Group	ypo3237; s376	ypo1821; s1427	ypo2837; s357	ypo1016; s1333	ypo3726; s949	ypo3098; s860	ypo3878; s968	ypo0829; s272	ypo0064; s447	No. (%) of strains
**1**	G	A	A	A	T	T	A	G	C	22 (36)
**2**	G	A	A	A^b^	G	C	C	G	C	39 (64)
**UG05-0454**	G	A	A	A	T	T	A	T	T	Der. control
**Antiqua**	A	G	G	C	G	C	C	G	C	Anc. control
CO92 Position	3602775	2067815	3166303	1148902	4171391	3456788	4353674	910389	75697	

^a^CO92 gene; SNP designation from [14].

^b^This SNP could not be amplified from 3 strains.

### PFGE

**Fig 2:**
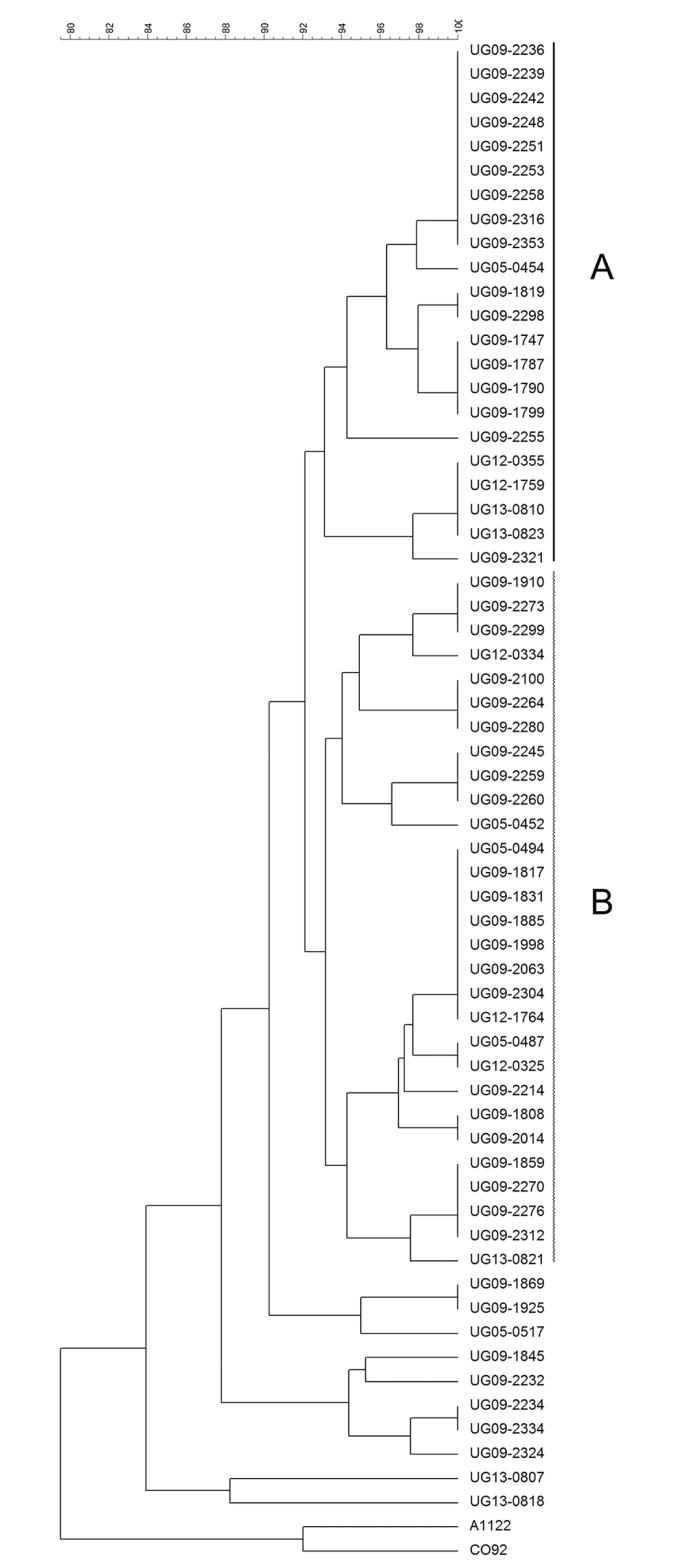
Cluster analysis of PFGE patterns for *Y*. *pestis* isolates from the West Nile region of Uganda. UPGMA of AscI PFGE patterns for 61 *Y*. *pestis* strains isolated from human plague cases in the West Nile region of Uganda. PFGE groups A (solid line) and B (dashed line) are indicated. *Y*. *pestis* CO92 and A1122 strains (1.ORI: North America) were included as outgroups.

**Fig 3:**
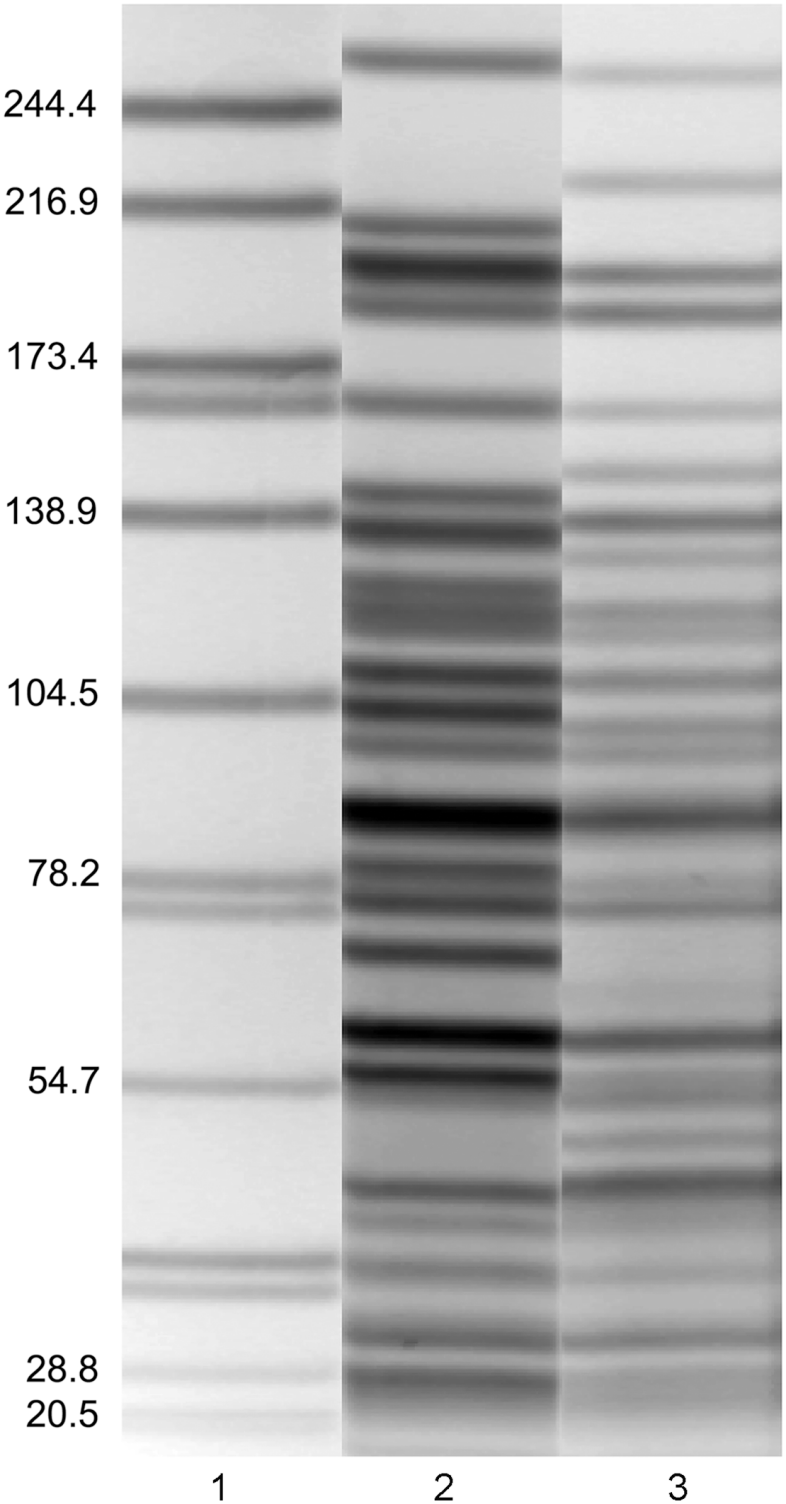
AscI PFGE patterns for *Y*. *pestis* isolates from the West Nile region of Uganda. Lane (1) *Salmonella braenderup* H9812 molecular marker in kilobases. Lane (2) PFGE Group A pattern. Lane (3) PFGE Group B pattern.

PFGE typing (using AscI) of the 61 *Y*. *pestis* isolates yielded multiple PFGE patterns (Fig 2). The majority of isolates (84%) clustered into two PFGE groups, termed Group A and Group B, which were differentiated from each other by at least 5 band differences (Fig 3). The remaining ten isolates did not cluster together and most displayed PFGE patterns with a higher molecular weight *Asc*I fragment as compared to PFGE patterns for isolates in Group A and Group B. Among isolates in Groups A and B, differences in PFGE patterns were also observed, although these differences were primarily limited to a single band. Comparison of groups assigned by SNPs and PFGE indicated that all isolates assigned to SNP Group 1 were classified as PFGE Group A. Isolates assigned to SNP Group 2 included the 29 isolates from PFGE Group B as well as the 10 additional isolates that did not fall into PFGE Group A or B.

The diversity of PFGE patterns observed among the Ugandan *Y*. *pestis* strains prompted an *in vitro* parallel passage experiment to determine if *Asc*I loci were rapidly changing. Comparison of PFGE patterns for 100 clones, representing 10,000 generations of the isolate UG09-2299, demonstrated no differences between the PFGE patterns, indicating no chromosomal changes affected these loci over the time frame analyzed. The mutation rate frequency of *Asc*I loci was calculated to be >10^−4^ (100 clonal lineages x 4 passages x 25.26 generations/colony).

### MLVA

**Fig 4:**
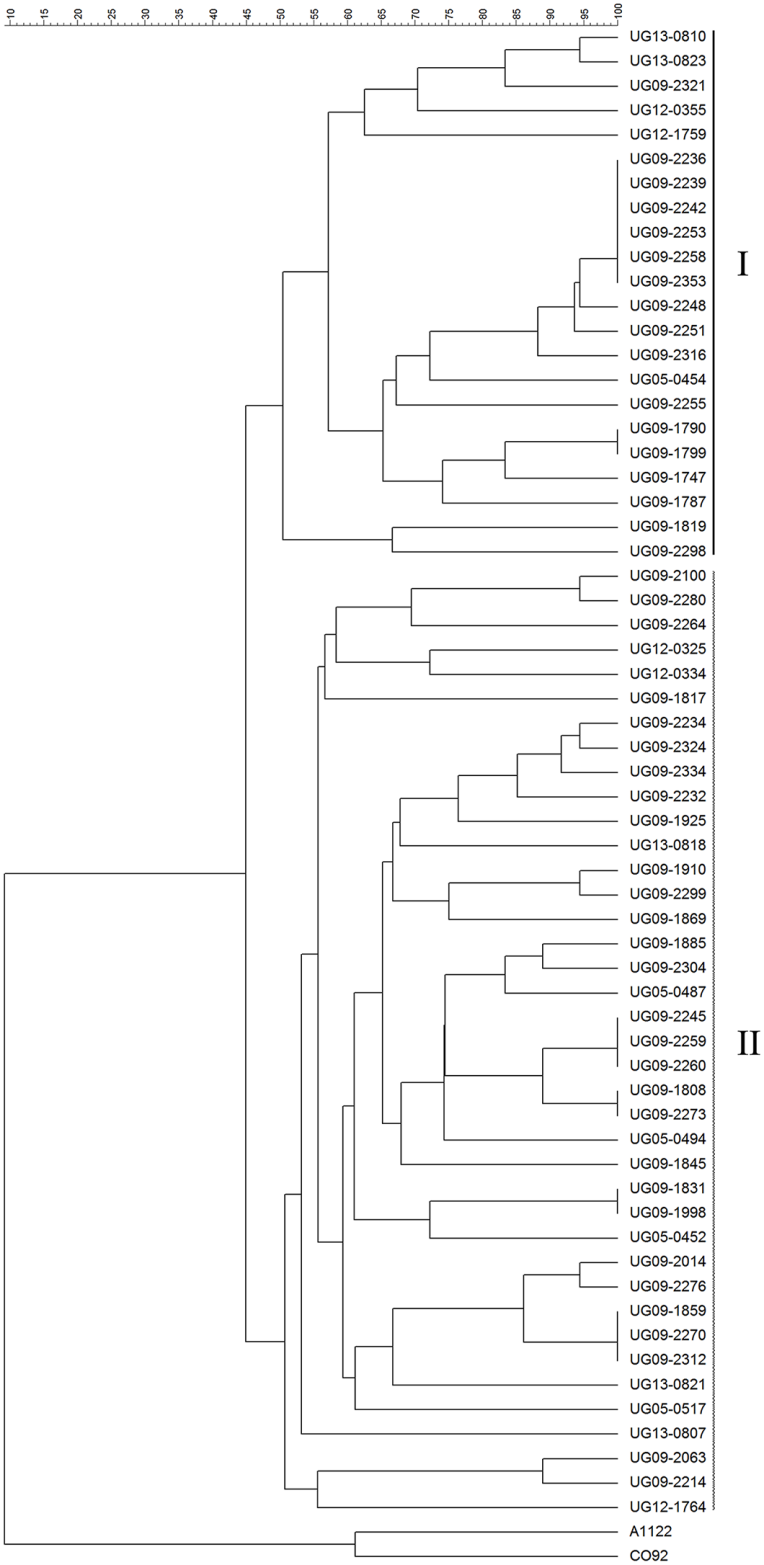
VNTR analysis of *Y*. *pestis* isolates from the West Nile region of Uganda. UPGMA based on 18 VNTRs in the 61 *Y*. *pestis* strains. MLVA groups I (solid line) and II (dashed line) are indicated. *Y*. *pestis* CO92 and A1122 strains (1.ORI: North America) were included as outgroups.

Eighteen VNTR loci were chosen to encompass the entire chromosome and to include a mixture of slow, intermediate, and rapidly changing loci. Among the 61 isolates, 16 of the VNTR loci (M12, M18, M19, M21, M22, M23, M25, M27, M28, M29, M31, M34, M58, M59, M68, M79) were found to be polymorphic while 2 markers (M24 and M33) showed no changes in repeat number (S2 Table). The number of repeats varied among the VNTR loci, ranging from 0 to 17 repeats as compared to the CO92 reference strain. Cluster analysis based on the 18 VNTRs yielded two distinct groups, denoted Group I and Group II (Fig 4). Isolates assigned to MLVA Group I and II showed 100% correlation with those isolates in SNP Groups 1 and 2. As expected, resolution of individual isolates was achieved by MLVA (Fig 4).

### Epidemiologic analysis

Overall, most culture-confirmed plague cases (88%) occurred during the months of October through December. The median age of source patients was 13 years (range: 3 years-65 years); most patients (61%) were female. The majority of infections (84%) were bubonic; overall plague mortality was 34%. The month or year of illness onset did not differ among infections caused by strains within SNP Groups 1 and 2. Strains within SNP Group I and II were isolated throughout the entire 9 year period of the study, specifically in three of the five years in which cases were cultured confirmed (2004, 2008 and 2012). Likewise, age, sex, clinical form of disease, and illness outcome did not differ between infections caused by SNP Group 1 or 2.

**Fig 5:**
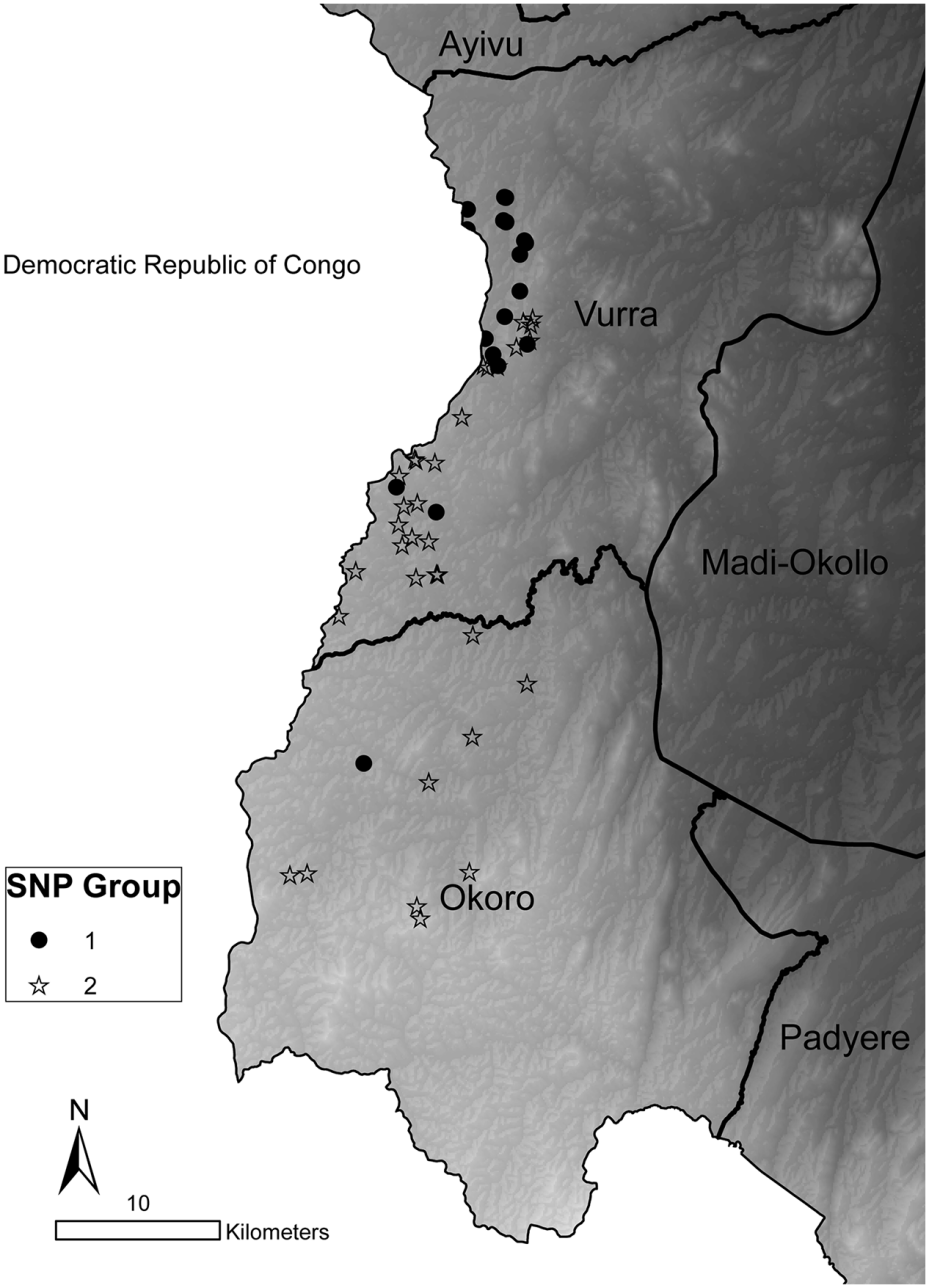
Geographic distribution of SNP genotypes. Map of Vurra and Okoro counties in the Arua and Zombo districts, respectively, in northwestern Uganda. *Y*. *pestis* isolates in SNP Groups 1 and 2 are shown. Isolates were plotted based on GPS coordinates of the source patient’s residence. Light to dark shading represents a high to low elevation gradient.

GPS coordinates corresponding to the source patients’ residence were available for 56 isolates (92%). Source patient residences’ mapped to an overall area of approximately 330 km^2^ (12 km across and 44 km long). Geographic distribution differed between infections caused by the two SNP groups. Group 1 strains were almost entirely within Vurra county, whereas Group 2 strains predominated in Okoro county (Fig 5). Furthermore, SNP Group 1 samples were from more northerly (median latitude: 2.8811°, range: 2.5955°-2.9064°) locations relative to SNP Group 2 (median latitude: 2.7197°, range: 2.5105°-2.8398°; Wilcoxon rank sums test, P<0.0001). Geographic separation of the two subpopulations was not absolute; overlap was observed in several areas (Fig 5). The observed geographic difference for the two genetic groups was paralleled by a significant difference in the elevation at which the source patients resided. The median elevation for Group 1 was 1330 meters (range: 1272–1496) as compared to 1442 meters (range: 1342–1608) for Group 2 (Wilcoxon rank sum test, P <0.0001).

## Discussion

Here, we investigated the diversity and molecular epidemiology of *Y*. *pestis* strains causing human infection in the West Nile region of Uganda. All 61 isolates recovered over the 9 year time period (2004 through 2012) fell into the 1.ANT lineage. Strains belonging to this lineage were previously shown to be geographically restricted to East and Central Africa and evolution of the 1.ANT lineage estimated to have occurred 628–6,914 years ago [14]. Three independent typing methods, SNPs, MLVA and PFGE, revealed that human plague in the West Nile region of Uganda is caused by at least two distinct 1.ANT genetic subpopulations, which are largely segregated by elevation. The two *Y*. *pestis* subpopulations persisted through the 9 year period consistent with their maintenance in highly localized enzootic cycles.

Source patients whose infections were due to SNP Group 1 strains lived at significantly lower elevations and further north as compared to those patients whose infections were due to SNP Group 2 strains, consistent with the relative independence of the two *Y*. *pestis* subpopulations, despite being localized to an overall area of only 330 km^2^. Of note, plague cases in Uganda were recorded only from Okoro county in Nebbi district for much of the 20^th^ century, until 1989, when cases were first noted further north in the Arua district [29]. It’s therefore possible the more northern Group 1 subpopulation represents a recent emergence.

We hypothesize the genetic differences between the two *Y*. *pestis* subpopulations may be associated with maintenance of the two subpopulations in different foci involving enzootic cycles with differing vector-host community composition. Subtle differences in vector community composition were observed previously between higher elevation sites in Zombo compared with lower elevation sites in Arua [4]. *Xenopsylla cheopis* was commonly encountered in the lower elevation sites, but rare or absent at higher elevations where it was replaced by *X*. *brasiliensis*, a finding that was consistent across studies [4,10]. Typing of *Y*. *pestis* strains isolated from small mammals and fleas across an elevation gradient will be important for determining what host and vector species are involved in enzootic maintenance of the two subpopulations. Additionally, as small mammals and fleas have considerably smaller home-ranges compared with humans, *Y*. *pestis* strains from these hosts may better delineate the geographic boundaries of the two genetic subpopulations.

Notably, the three typing methods used here, SNPs, PFGE and MLVA, all identified two genetic subpopulations, even though each method recognizes different mutation types in the *Y*. *pestis* genome (i.e. single nucleotide variation, tandem repeats, insertion sequence (IS) mediated rearrangements). Although previous studies have shown correlation between strain groupings by SNPs and MLVA [24,30], the links detected here between PFGE and SNPs and PFGE and MLVA were not expected. Genome sequencing of *Y*. *pestis* isolates with distinct FseI PFGE patterns previously demonstrated that PFGE band differences were due to large-scale IS element mediated genome rearrangements (different order and orientation of genome segments) [20]. With the rapid rise in the number of assembled *Y*. *pestis* genomes, large-scale rearrangements mediated by IS elements has become an emerging theme in this pathogen [18,20,31,32]. It is therefore likely that two *Y*. *pestis* subpopulations identified by AscI PFGE have rearranged genome orders with respect to one another (e.g., 5 band difference) and that other genomic rearrangements, as evidenced by AscI PFGE band differences, are present within each of the two subpopulations. A correlation between large-scale genome rearrangement and increased nucleotide variation localized to rearrangement breakpoints, has recently been shown in several other prokaryotes, including *Burkholderia*, *Pseudomonas* and *Shigella* [33]. Genome sequencing and assembly of *Y*. *pestis* strains encompassing the two West Nile subpopulations will be important for determining if increased nucleotide variation may also be observed near genome rearrangements in *Y*. *pestis*, thus explaining the correlation between typing methods that detect differences in nucleotides or genome structure.

Previous analyses of *Y*. *pestis* and other bacterial vector-borne zoonotic pathogens have demonstrated the presence of multiple strains and subpopulations within a defined, localized geographical region [20,34]. The finding that human plague occurring in a limited geographic area of Uganda is also caused by distinct genetic subpopulations of *Y*. *pestis*, is consistent with genomic diversification of *Y*. *pestis* at small geographic scales. The identification and characterization of *Y*. *pestis* subpopulations causing human plague in the West Nile region will be useful for ongoing efforts to identify ecologic and environmental factors associated with elevated plague risk.

## Supporting Information

S1 Table Melt-MAMA primer sequences. (PDF)

S2 Table SNP and VNTR data. (PDF)
