# Supplementary material for: Two Distinct Yersinia pestis Populations Causing Plague among Humans in the West Nile Region of Uganda
Source: PLoS Negl Trop Dis. 2016 Feb 11;10(2):e0004360. doi: 10.1371/journal.pntd.0004360 (PMC4750964; doi:10.1371/journal.pntd.0004360)
Supplement: S1 Table — (PDF) [file pntd.0004360.s001.pdf]

S1 Table. Melt-MAMA primer sequences.

	Melt-MAMA Primer (5'→ 3')		
**CO92 Gene**	**Ancestral**	**Derived**	**Common**
**ypo3237**	GATTTCATCGCATAAGCTACG	CGGCGGCGGCGGCGGGATTTCATCGCATAAGCTCCA	AGCAGTTGTCGGTGTTCGT
**ypo1821**	ATCACCACCCGCTCCCA	CGGCGGCGGCGGCGGATCACCACCCGCTCTCG	CGGCGGTTGGGGTTTG
**ypo2837**	CCCTGTTTGCTGAACGTATG	CGGCGGCGGCGGCGGCCCCTGTTTGCTGAACGTCTA	GATAACCTCGGCGGCAC
**ypo1016**	GGCATTGCATGGCACTTC	CGGCGGCGGCGGCGGGGCATTGCATGGCACCTA	GAGCGACCCAAGCGGA
**ypo3726**	GCTCGTCTTGTGTTGTAATGCT	CGGCGGCGGCGGCGGCGCTCGTCTTGTGTTGTAATCCG	CGCAGAGCGTTTTTGATCG
**ypo3098**	CATTTTTTGCGACATACTGTAACTT	CGGCGGCGGCGGCGGCATTTTTTGCGACATACTGTAACTC	CCCTATCTGATATTGTAGAAACCTT
**ypo3878**	TAACCTTGTTTTGACAGGAAAAGAC	GCCGCCGCCGCCGCCGTAACCTTGTTTTGACAGGAAAATAA	AACTTGAGACATAGGCGCGA
**ypo0829**	GCATTTCATCCGGGGAT	CGGCGGCGGCGGCGGCGCATTTCATCCGGGCAG	GGCTCAGGCCGTAGTAC
**ypo0064**	AAATTCATAAAGATTAATGTATCCCT	GCCGCCGCCGCGGGCGAAATTCATAAAGATTAATGTATCGCC	AACCAACCGTGATTCAAGCAA
