# Supplementary material for: Two Distinct Yersinia pestis Populations Causing Plague among Humans in the West Nile Region of Uganda
Source: PLoS Negl Trop Dis. 2016 Feb 11;10(2):e0004360. doi: 10.1371/journal.pntd.0004360 (PMC4750964; doi:10.1371/journal.pntd.0004360)
Supplement: S2 Table — (PDF) [file pntd.0004360.s002.pdf]

S2 Table. SNP and VNTR data.

	SNP[a]										18-locus VNTR																		
Strain	ypo3237	ypo1821	ypo2837	ypo1016	ypo3726	ypo3098	ypo3878	ypo0829	ypo0064	SNP Group	M68	M23	M22	M21	M12	M18	M24	M33	M34	M31	M19	M28	M59	M58	M29	M79	M25	M27	MLVA Group
UG13-0810	G	A	A	-[b]	T	T	A	G	C	1	0	-1	-1	2	5	2	-3	4	7	3	-17	-3	-1	-3	-4	-6	-13	1	I
UG13-0823	G	A	A	-[b]	T	T	A	G	C	1	0	-1	-1	2	5	2	-3	4	7	3	-17	-3	-1	-3	-4	-6	-13	0	I
UG09-2321	G	A	A	A	T	T	A	G	C	1	0	-1	-1	2	5	3	-3	4	7	3	-15	-3	-1	-3	-4	-6	-13	3	I
UG12-0355	G	A	A	-[b]	T	T	A	G	C	1	0	-1	2	3	4	2	-3	4	7	4	-17	-3	-1	-3	-4	-6	-13	0	I
UG12-1759	G	A	A	A	T	T	A	G	C	1	0	-2	-1	2	4	2	-3	4	7	3	-17	-2	-1	-3	-4	-5	-12	1	I
UG09-2236	G	A	A	A	T	T	A	G	C	1	0	-1	0	-1	5	3	-3	4	7	2	-16	-3	-1	-3	-4	-6	-12	2	I
UG09-2239	G	A	A	A	T	T	A	G	C	1	0	-1	0	-1	5	3	-3	4	7	2	-16	-3	-1	-3	-4	-6	-12	2	I
UG09-2242	G	A	A	A	T	T	A	G	C	1	0	-1	0	-1	5	3	-3	4	7	2	-16	-3	-1	-3	-4	-6	-12	2	I
UG09-2253	G	A	A	A	T	T	A	G	C	1	0	-1	0	-1	5	3	-3	4	7	2	-16	-3	-1	-3	-4	-6	-12	2	I
UG09-2258	G	A	A	A	T	T	A	G	C	1	0	-1	0	-1	5	3	-3	4	7	2	-16	-3	-1	-3	-4	-6	-12	2	I
UG09-2353	G	A	A	A	T	T	A	G	C	1	0	-1	0	-1	5	3	-3	4	7	2	-16	-3	-1	-3	-4	-6	-12	2	I
UG09-2248	G	A	A	A	T	T	A	G	C	1	0	-1	0	-1	5	3	-3	4	5	2	-16	-3	-1	-3	-4	-6	-12	2	I
UG09-2251	G	A	A	A	T	T	A	G	C	1	0	-1	0	-1	5	3	-3	4	7	2	-16	-3	-1	-3	-4	-6	-12	3	I
UG09-2316	G	A	A	A	T	T	A	G	C	1	0	-1	0	3	5	3	-3	4	7	2	-16	-3	-1	-3	-4	-6	-12	0	I
UG05-0454	G	A	A	A	T	T	A	T	T	1	0	-1	0	2	5	4	-3	4	7	3	-16	-2	-1	-3	-4	-6	-12	0	I
UG09-2255	G	A	A	A	T	T	A	G	C	1	0	-1	0	2	4	3	-3	4	6	1	-16	-3	-1	-3	-4	-6	-11	0	I
UG09-1790	G	A	A	A	T	T	A	G	C	1	0	-1	1	2	5	3	-3	4	8	3	-16	-3	-1	-3	-5	-6	-11	2	I
UG09-1799	G	A	A	A	T	T	A	G	C	1	0	-1	1	2	5	3	-3	4	8	3	-16	-3	-1	-3	-5	-6	-11	2	I
UG09-1747	G	A	A	A	T	T	A	G	C	1	0	-1	1	2	4	3	-3	4	8	3	-16	-3	-1	-3	-5	-6	-12	1	I
UG09-1787	G	A	A	A	T	T	A	G	C	1	0	-1	1	0	5	3	-3	4	7	2	-16	-3	-1	-3	-5	-6	-11	7	I
UG09-1819	G	A	A	A	T	T	A	G	C	1	0	-1	1	2	7	1	-3	4	8	1	-15	-3	-1	-3	-4	-7	-12	1	I
UG09-2298	G	A	A	A	T	T	A	G	C	1	0	-1	1	3	3	1	-3	4	5	1	-16	-2	-1	-3	-4	-7	-12	0	I
UG09-2100	G	A	A	A	G	C	C	G	C	2	0	3	2	1	4	8	-3	4	5	2	-12	-3	-1	-3	-4	-6	-9	-3	II
UG09-2280	G	A	A	A	G	C	C	G	C	2	0	3	2	1	4	8	-3	4	5	3	-12	-3	-1	-3	-4	-6	-9	-3	II
UG09-2264	G	A	A	A	G	C	C	G	C	2	0	5	2	1	5	6	-3	4	4	2	-12	-3	-1	-3	-4	-6	-9	0	II
UG12-0325	G	A	A	A	G	C	C	G	C	2	0	4	1	0	2	8	-3	4	5	3	-13	-3	-1	-3	-4	-6	-9	-2	II
UG12-0334	G	A	A	A	G	C	C	G	C	2	0	1	1	0	2	4	-3	4	5	4	-14	-3	-1	-3	-4	-6	-9	-3	II
UG09-1817	G	A	A	A	G	C	C	G	C	2	0	5	2	0	2	9	-3	4	1	2	-14	-3	-1	-3	-3	-6	-9	-5	II
UG09-2234	G	A	A	A	G	C	C	G	C	2	0	6	4	1	0	6	-3	4	3	2	-13	-3	-1	-3	-4	-6	-9	-5	II
UG09-2324	G	A	A	A	G	C	C	G	C	2	0	6	4	1	0	6	-3	4	3	2	-13	-3	-1	-3	-4	-7	-9	-5	II
UG09-2334	G	A	A	A	G	C	C	G	C	2	0	6	4	1	0	6	-3	4	3	2	-13	-3	-1	-3	-4	-6	-9	-4	II
UG09-2232	G	A	A	A	G	C	C	G	C	2	0	6	4	1	3	5	-3	4	3	2	-13	-3	-1	-3	-4	-6	-9	-5	II
UG09-1925	G	A	A	A	G	C	C	G	C	2	0	3	4	0	3	3	-3	4	3	2	-13	-3	-1	-3	-4	-6	-9	-5	II
UG13-0818	G	A	A	A	G	C	C	G	C	2	0	5	2	1	0	7	-3	4	3	2	-13	-3	-1	-3	-4	-6	-10	-6	II
UG09-1910	G	A	A	A	G	C	C	G	C	2	0	4	3	1	3	6	-3	4	3	2	-15	-3	-1	-3	-4	-6	-10	-4	II
UG09-2299	G	A	A	A	G	C	C	G	C	2	0	4	3	1	3	6	-3	4	3	2	-15	-3	-1	-3	-4	-6	-10	-5	II
UG09-1869	G	A	A	A	G	C	C	G	C	2	0	5	3	1	3	6	-3	4	3	1	-13	-3	-1	-3	-4	-6	-8	-4	II
UG09-1885	G	A	A	A	G	C	C	G	C	2	0	4	3	2	1	11	-3	4	4	1	-16	-3	-1	-3	-4	-6	-9	-5	II
UG09-2304	G	A	A	A	G	C	C	G	C	2	0	4	3	2	0	11	-3	4	4	2	-16	-3	-1	-3	-4	-6	-9	-5	II
UG05-0487	G	A	A	A	G	C	C	G	C	2	0	4	3	2	1	9	-3	4	4	2	-14	-3	-1	-3	-4	-6	-9	-5	II
UG09-2245	G	A	A	A	G	C	C	G	C	2	0	4	3	1	0	7	-3	4	3	2	-16	-3	-1	-3	-4	-6	-9	-5	II
UG09-2259	G	A	A	A	G	C	C	G	C	2	0	4	3	1	0	7	-3	4	3	2	-16	-3	-1	-3	-4	-6	-9	-5	II
UG09-2260	G	A	A	A	G	C	C	G	C	2	0	4	3	1	0	7	-3	4	3	2	-16	-3	-1	-3	-4	-6	-9	-5	II
UG09-1808	G	A	A	A	G	C	C	G	C	2	0	2	3	1	1	7	-3	4	3	2	-16	-3	-1	-3	-4	-6	-9	-5	II
UG09-2273	G	A	A	A	G	C	C	G	C	2	0	2	3	1	1	7	-3	4	3	2	-16	-3	-1	-3	-4	-6	-9	-5	II
UG05-0494	G	A	A	A	G	C	C	G	C	2	0	3	3	1	1	6	-3	4	4	2	-17	-3	-1	-3	-4	-6	-9	-5	II
UG09-1845	G	A	A	A	G	C	C	G	C	2	0	5	3	1	-1	7	-3	4	5	0	-13	-3	-1	-3	-4	-6	-9	-5	II
UG09-1831	G	A	A	A	G	C	C	G	C	2	0	3	3	1	3	5	-3	4	4	1	-14	-3	-1	-3	-4	-6	-8	-5	II
UG09-1998	G	A	A	A	G	C	C	G	C	2	0	3	3	1	3	5	-3	4	4	1	-14	-3	-1	-3	-4	-6	-8	-5	II
UG05-0452	G	A	A	A	G	C	C	G	C	2	0	3	1	1	3	5	-3	4	2	2	-14	-3	-1	-3	-4	-6	-9	0	II
UG09-2014	G	A	A	A	G	C	C	G	C	2	0	4	3	1	2	9	-3	4	4	0	-12	-3	-1	-3	-4	-6	-9	-4	II
UG09-2276	G	A	A	A	G	C	C	G	C	2	0	4	3	1	2	9	-3	4	4	0	-12	-3	-1	-3	-4	-6	-9	-5	II
UG09-1859	G	A	A	A	G	C	C	G	C	2	0	4	3	2	2	9	-3	4	4	-1	-12	-3	-1	-3	-4	-6	-9	-4	II
UG09-2270	G	A	A	A	G	C	C	G	C	2	0	4	3	2	2	9	-3	4	4	-1	-12	-3	-1	-3	-4	-6	-9	-4	II
UG09-2312	G	A	A	A	G	C	C	G	C	2	0	4	3	2	2	9	-3	4	4	-1	-12	-3	-1	-3	-4	-6	-9	-4	II
UG13-0821	G	A	A	A	G	C	C	G	C	2	0	4	0	1	2	9	-3	4	3	0	-14	-3	-2	-3	-4	-6	-9	-5	II
UG05-0517	G	A	A	A	G	C	C	G	C	2	-1	2	3	2	2	2	-3	4	3	2	-16	-3	-1	-3	-4	-6	-9	-4	II
UG13-0807	G	A	A	A	G	C	C	G	C	2	0	1	3	2	1	9	-3	4	2	1	-16	-3	-1	-3	-4	-6	-8	-6	II
UG09-2063	G	A	A	A	G	C	C	G	C	2	0	3	7	2	2	8	-3	4	3	4	-14	-3	-1	-3	-4	-4	-9	-3	II
UG09-2214	G	A	A	A	G	C	C	G	C	2	0	3	7	2	2	7	-3	4	3	5	-14	-3	-1	-3	-4	-4	-9	-3	II
UG12-1764	G	A	A	A	G	C	C	G	C	2	0	3	0	1	2	10	-3	4	3	1	-13	-3	-1	-4	-4	-5	-9	-6	II

[a]CO92 gene.

[b]This SNP could not be amplified from 3 strains.
